# The infectious particle of insect-borne totivirus-like Omono River virus has raised ridges and lacks fibre complexes

**DOI:** 10.1038/srep33170

**Published:** 2016-09-12

**Authors:** Kenta Okamoto, Naoyuki Miyazaki, Daniel S. D. Larsson, Daisuke Kobayashi, Martin Svenda, Kerstin Mühlig, Filipe R. N. C. Maia, Laura H. Gunn, Haruhiko Isawa, Mutsuo Kobayashi, Kyoko Sawabe, Kazuyoshi Murata, Janos Hajdu

**Affiliations:** 1 The Laboratory of Molecular Biophysics, Department of Cell and Molecular biology, Uppsala University, Sweden; 2 National Institute for Physiological Sciences (NIPS), Japan; 3 Department of Medical Entomology, National Institute of Infectious Diseases (NIID), Japan

## Abstract

*Omono River virus* (OmRV) is a double-stranded RNA virus isolated from Culex mosquitos, and it belongs to a group of unassigned insect viruses that appear to be related to Totiviridae. This paper describes electron cryo-microscopy (cryoEM) structures for the intact OmRV virion to 8.9 Å resolution and the structure of the empty virus-like-particle, that lacks RNA, to 8.3 Å resolution. The icosahedral capsid contains 120-subunits and resembles another closely related arthropod-borne totivirus-like virus, the infectious myonecrosis virus (IMNV) from shrimps. Both viruses have an elevated plateau around their icosahedral 5-fold axes, surrounded by a deep canyon. Sequence and structural analysis suggests that this plateau region is mainly composed of the extended C-terminal region of the capsid proteins. In contrast to IMNV, the infectious form of OmRV lacks extensive fibre complexes at its 5-fold axes as directly confirmed by a contrast-enhancement technique, using Zernike phase-contrast cryo-EM. Instead, these fibre complexes are replaced by a short “plug” structure at the five-fold axes of OmRV. OmRV and IMNV have acquired an extracellular phase, and the structures at the five-fold axes may be significant in adaptation to cell-to-cell transmission in metazoan hosts.

Icosahedral dsRNA viruses have tremendous impact on food production, animal welfare, and human health^1^,^2^,^3^,^4^,^5^,^6^. Arthropod hosts such as insects are known to be important transmitters of dsRNA viruses^3^,^7^,^8^. Ten distinct families of the dsRNA viruses have been established so far: *Birnaviridae*, *Botybirnaviridae*, *Chrysoviridae*, *Cystoviridae*, *Megabirnaviridae*, *Partitiviridae*, *Picobirnaviridae*, *Quadriviridae*, *Reoviridae*, and *Totiviridae*^9^. Most dsRNA viruses share a similar capsid architecture with a T = 1 capsid lattice that comprises 120 chemically identical capsid proteins (CPs)^9^, except for *Birnaviridae*^10^ and *Chrysoviridae*^11^. The icosahedral asymmetric unit of the T = 1 lattice consists of a dimer of two CPs. Each CP adopts a conserved α + β fold that is enriched in α-helices^12^,^13^. The conserved capsid geometry of dsRNA viruses implies a common origin of dsRNA viruses and reflects fundamental requirements for their survival strategies such as replication and propagation^10^,^13^,^14^.

Totiviruses, partitiviruses and chrysoviruses primarily infect prokaryotes and primitive eukaryotes, such as protozoa and fungi, while partiviruses and reoviruses infect a broad host range from prokaryotes to higher eukaryotes, including plants and vertebrates^9^. The fungal and protozoan dsRNA viruses propagate only intracellularly: vertically due to the frequent cell division of the primitive hosts^15^,^16^,^17^ or upon cell fusion, e.g. hyphal anastomosis during mating^15^. However, dsRNA viruses that infect higher eukaryotes have an extracellular phase in their life cycles and thus require intercellular transmission mechanisms, such as cell attachment and entry, for propagation. In the case of the reoviruses, an additional T = 13 outer capsid has been acquired that encloses the conserved T = 1 inner capsid^18^,^19^. The outer capsid proteins provide cell binding and membrane penetration functions^20^,^21^. On the other hand, the picobirnavirus (*Rabbit picobirnavirus*; RaPBV) has a single-layered T = 1 capsid. The capsid protein has a protruding domain that forms arch-like protrusions on the viral surface and likely facilitates binding to receptor molecules on the host cell surface^22^. Available data indicate that dsRNA viruses most commonly attain cell-binding capability by specialized capsid surface structures.

*Totiviridae*, a viral family of dsRNA viruses, has five taxonomically approved genera (*Totivirus*, *Victorivirus*, *Giardiavirus*, *Leishmaniavirus* and *Trichomonasvirus*). These all infect primitive eukaryotes such as fungi, parasites and protozoa^23^. Recently a handful of related, but yet unassigned, metazoan totivirus-like viruses have been reported. *Infectious myonecrosis virus* (IMNV) was isolated from the skeletal muscle of shrimp (*Litopenaeus vannamei*) in Brazil in 2003^3^,^24^. *Piscine myocarditis virus* (PMCV) was identified as the causative agent for cardiomyopathy syndrome in salmon farms in Norway^25^. *Drosophila totivirus* (DTV) and *Armigeres subalbatus totivirus* (AsTV) were found in S2-GMR *Drosophila* cells and in *Armigeres* mosquitoes, respectively^26^,^27^. *Omono River virus* (OmRV) was recently isolated from *Culex* mosquitoes in northern Japan^28^. All the above metazoan totivirus-like viruses cluster next to *Giardia lamblia virus* (GLV) as a monophyletic group in the *Totiviridae* tree, and thus the most recent proposal for resolving the taxonomical quandary is to classify them together with GLV as a distinct family or as a subfamily within the family *Totiviridae*^29^ (see Supplementary Fig. S1).

Four fungal and protozoan totiviruses have been structurally determined: *Saccharomyces virus L-A* (ScV-L-A)^12^, *Helminthosporium victoriae 190S virus* (Hv190SV)^30^, *Trichomonas vaginalis virus 1* (TVV1)^31^ and GLV^29^. These protozoan and fungal totiviruses have an archetypical T = 1 capsid composed of 60 asymmetric CP dimers with diameters of 40–50 nm and do not have projections on their surfaces. Conserved α-helices + β-sheets (α + β) and connected β-sheet rich (β-rich) domains have been observed in their capsid structures. In addition to these fungal and protozoan totiviruses one 3D structure of a metazoan totivirus-like virus, IMNV, has been determined so far. The IMNV structure reveals fibres extending from the icosahedral 5-fold axes that represent minor virus proteins^30^,^31^,^32^,^33^. These fibrous structures resemble those of other metazoan dsRNA viruses, which are important for cell entry^34^. However, it remains unclear whether the IMNV protrusions are involved in cell attachment and entry and if such protrusion structures are common in other metazoan totivirus-like viruses.

We present here the first 3D structure of an insect totivirus-like virus, OmRV, solved by cryo-EM and 3D reconstruction. The results are in line with the evolutionary acquisition of extracellular transmission capability and host specificity of totivirus-like dsRNA viruses. Furthermore, we performed proteomic, infection and sequence studies to trace the role of minor structural proteins in the virion. These results reveal that the pores on the icosahedral 5-fold axes in the infectious form of OmRV are obstructed and the virus has no extensive fibre structures. The absence of the fibres was confirmed by Zernike phase contrast cryoEM (ZPC-cryoEM), a technique commonly used for detecting low-contrast structural features of viruses^35^,^36^,^37^,^38^. This is in contrast to results from IMNV^32^ where the icosahedral 5-fold axes hold prominent fibre complexes on the viral surface. OmRV also had a raised ridge presumably consisting of the extra C-terminal α-helix rich domain (α-rich) of the major CP around the icosahedral 5-fold axis. The ridge forms the walls of a canyon, which encirculates the 5-fold axis. These features are conserved amongst the arthropod totivirus-like viruses that infect by extracellular means.

## Results

### Identification of OmRV structural proteins

**Figure 1:**
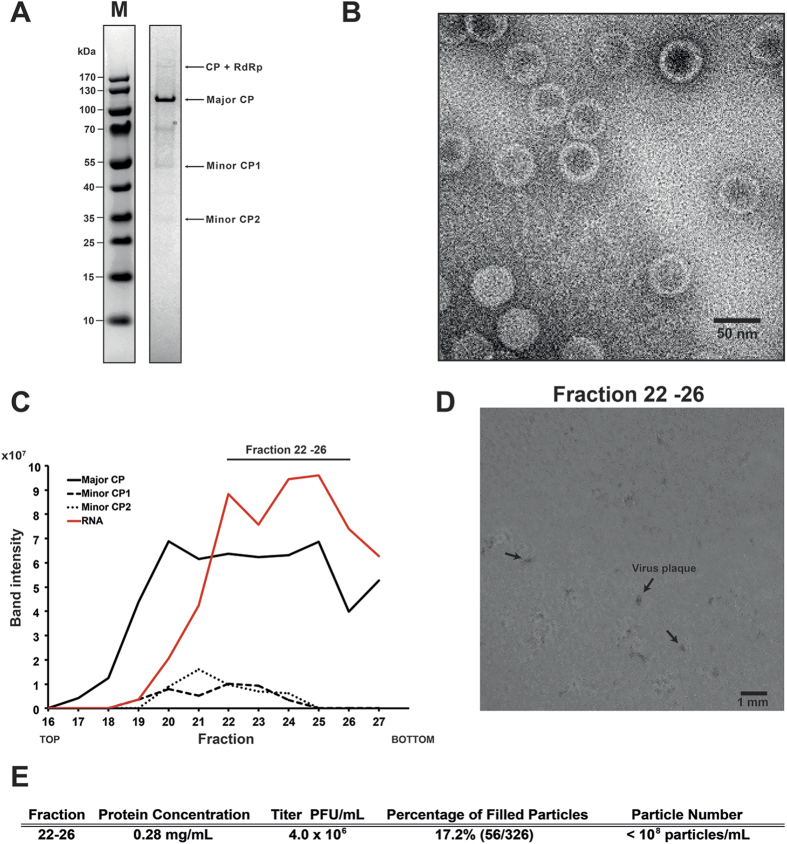
Sample preparation and biochemical assays for cryo-EM. (**A**) SDS-PAGE of purified OmRV. Arrows indicate the major coat protein (CP) band and other minor OmRV protein bands. The composition of the protein bands were further investigated by tandem-MS spectrometry (see Supplementary Fig. S2). An asterisk indicates a background band. (**B**) TEM image of purified and negatively stained OmRV particles. (**C**) Protein and dsRNA content in the sucrose gradient fractions obtained during OmRV purification. The fraction numbers represent 500 μL fractions numbered from top to bottom. The intensities of the protein and dsRNA bands were measured by a densitometer and plotted along the fractions. Peak fractions 22–26 were pooled and adjusted to 0.28 mg/mL total protein concentration for use in the infectivity assays shown in (**D**,**E**). (**D**) Plaques (black arrows) in C6/36 *A. albopictus* mosquito cells observed three days post-infection with fraction 22–26. (**E**) Infectivity titre and full particle ratio in fraction 22–26. The number of full and empty particles was counted from cryo-EM images of fraction 22–26 (see Supplementary Fig. S3). The total particle number in the peak fractions after 10 times dilution was lower than the detection range of NTS (10^7^ particles/mL) and therefore the total particle number in the original fraction was estimated to be less than 10^8^ particles/mL.

The OmRV has two open reading frames (ORFs) encoding for the CP and the RNA-dependent RNA polymerase (RdRp)^28^. SDS-PAGE analysis of purified virus fractions indicated that the OmRV virion is composed of one major protein (the most abundant protein in the virus particle, roughly 100 kDa in size) and three minor proteins (>170 kDa, ~55 kDa, and ~30 kDa) (Fig. 1A), consistent with a previous report^28^. In this study, the protein composition was further analysed by tandem mass (MS/MS) spectrometry (see Supplementary Fig. S2). The ~100 kDa protein corresponds to the OmRV major CP. From the ~100 kDa band, almost continuous coverage was obtained from the predicted 897 aa (amino acid) (96.8 kDa) major CP with peptides starting from the sequence FDQDR to the C-terminus (aa positions 841–1685 in ORF1, 845 aa in total) (see Supplementary Fig. S2). However, no N-terminal peptides of ORF1 were detected in the ~100 kDa band. The minor >170 kDa protein band was expected to be a 192 kDa CP–RdRp fusion protein since a ribosomal -1 frameshift may cause the two open reading frames to be translated in tandem^28^. MS/MS spectrometry confirmed that the band contained peptides from both the CP and the RdRp (see Supplementary Fig. S2). From the ~55 kDa and ~30 kDa bands, several N-terminal peptides of ORF1 were identified (see Supplementary Fig. S2). Thus, the ~55 kDa and ~30 kDa proteins were designated as minor CP1 and minor CP2, respectively, although these minor proteins remain functionally uncharacterized. The stoichiometry of the minor proteins was estimated from the intensity and molecular weight of the bands (Fig. 1A, see Supplementary Fig. S2). The CP-RdRp was 20–35 fold less frequent than the major CP. Minor CP 1 and 2 were 7–10 and 3–5 fold less frequent, respectively.

The purified fraction in Fig. 1A was concentrated for use in structural analyses. Negative stain electron microscopy showed that the purified virus particles contained uniform spherical particles approximately 40 nm in diameter (Fig. 1B). Subsequently, cryo-EM was performed to determine the 3D structure.

### Biochemical composition and infectivity of full OmRV particles

Purified OmRV was further characterized on a more finely fractionated sucrose gradient (Fig. 1C). The major CP was detected in almost all fractions, while the minor proteins CP1 and CP2 were detected in fractions 20–24. Viral dsRNA was mainly detected in fractions 22–26 (Fig. 1C, see Supplementary Fig. S3). Minor CP abundance appeared to correlate with the amount of the major CP rather than dsRNA (Fig. 1C). The pooled 22–26 fraction (dsRNA-containing fractions) was used for further infectivity assays. The fraction was diluted 10^5^ times and inoculated onto C6/36 cells, which induced a clear cytopathic effect (CPE) three days post inoculation (see Supplementary Fig. S3). The plaques in the cell culture inoculated with fraction 22–26 were 0.1–0.2 mm in size (arrows in Fig. 1D). The infectivity titre gave a value of 4.0 × 10^6^ plaque forming units (PFU)/mL (Fig. 1E). The number of dsRNA-full and empty particles in fraction 22–26 was counted in raw cryo-EM images (see Supplementary Fig. S3). Consequently, the percentage of dsRNA-full particles was 17.2% in the total fractions (Fig. 1E). The total number of particles in the purified fractions was estimated to be less than 10^8^ particles/mL by nanoparticle tracking analysis (Fig. 1E).

Fraction 22–26 contained a large number of empty particles that were not able to form plaques in C6/36 cells because of their lack of a viral genome (Fig. 1E, see Supplementary Fig. S3). Assuming that all dsRNA-full particles are infectious, the total number of particles was 5–10 times higher than the number of PFUs (4.0 × 10^6^ PFU/mL) because only 17.2% of the particles in fraction 22–26 were filled with dsRNA, which gives a total of roughly 10^7^ particles/mL in the fraction (Fig. 1E). The total number of particles was estimated to be less than 10^8^ particles/mL using nano particle tracking system (Fig. 1E). Hence, both estimates agreed with a total particle count in the range 10^7^–10^8^ particles/mL. These results indicate that almost all dsRNA-full particles in the fraction were infectious to C6/36 mosquito cells.

### Cryo-EM and 3D reconstruction

**Figure 2:**
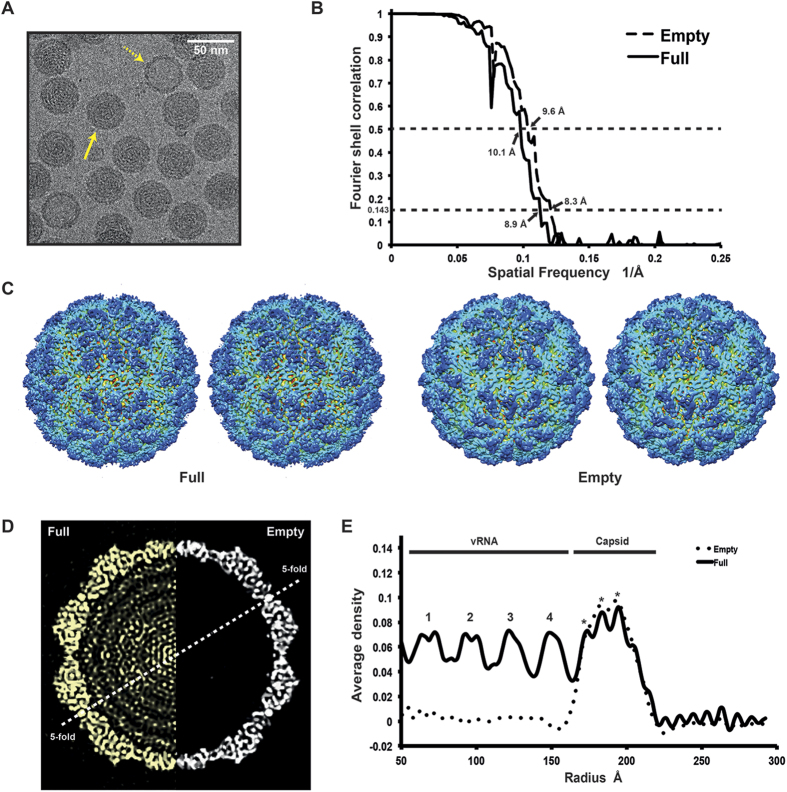
The 3D structure of empty and full OmRV particle. (**A**) Raw cryo-EM image of purified OmRV obtained at a nominal magnification of 50,000 with 200 kV accelerating voltage using defocus phase contrast (DPC) cryo-EM. Dotted and solid arrows indicate empty and full virus particles, respectively. The white scale bar represents 50 nm. (**B**) Resolution plot for the 3D reconstruction of OmRV. The resolution was calculated using the Fourier Shell Correlation (FSC) method of splitting each data set into two halves. The calculated spatial frequencies at 0.143 correlation was 1/8.9 (0.112) Å^−1^ and at 0.5 correlation was 1/10.1 (0.099) Å^−1^ for the full particles, and 1/8.3 (0.120) Å^−1^ and 1/9.6 (0.104) Å^−1^ for the empty particles. This gives a resolution of 8.9 Å (10.1 Å) and 8.3 Å (9.6 Å), respectively. (**C**) Stereo views of reconstructed full and empty OmRV structures. The full and empty structures were rendered at an isodensity contour level of 2.1 *σ* and 2.6 *σ*, respectively. (**D**) Cross-sections and (**E**) radially averaged density profiles of the full and empty particles.

Structural analysis of the purified OmRV was performed by cryo-EM. Electron micrographs show uniform spherical dsRNA-full and empty particles with a diameter of approximately 40 nm in underfocus contrast of 1 to 3 μm (Fig. 2A). The individual viral particles were sorted and classified into two distinct groups according to either strong or weak densities in the interior regions, which presumably represent particles with or without packaged viral dsRNA, respectively (Fig. 2A). The final resolutions of the reconstructed 3D structures of the empty and full particles were assessed to be 8.3 Å and 8.9 Å by the FSC method with a 0.143 cutoff (Fig. 2B)^39^. Stereo views of the reconstructed final models of dsRNA-full and empty particles are shown in Fig. 2C. Cross-sections of the dsRNA-full and empty OmRV particles are shown side by side in Fig. 2D. The reconstruction of the empty particles shows uniformly low electron density inside the capsid, while the full particles show concentric layers of density corresponding to the packed dsRNA (Fig. 2D,E). Similar features were also observed in IMNV^32^. The radially averaged electron density indicates that OmRV has a ~61 Å thick capsid monolayer with a maximum outer diameter of ~440 Å and an inner diameter of ~318 Å (Fig. 2E). The fine structure of this density seems to consist of three denser regions with peaks at radius 172 Å, 183 Å, and 193 Å, respectively (asterisks in Fig. 2E).

### Radial sections of the capsid and an equivalent surface view

**Figure 3:**
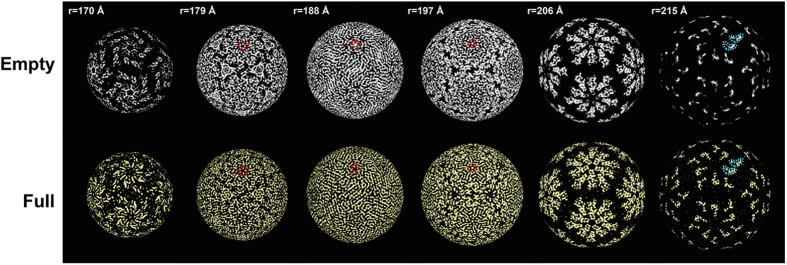
Radial sections of the empty and full OmRV particles. Radial sections in the 3D density of the empty (white) and full (yellow) particles, which are shown at radiuses of 170, 179, 188, 197, 206, and 215 Å. The boomerang-shaped raised ridge domains of subunits and the obstructed pore are outlined with light blue and red dotted lines, respectively. The full and empty structures were rendered at an isodensity contour level of 2.1 *σ* and 2.6 *σ*, respectively.

**Figure 4:**
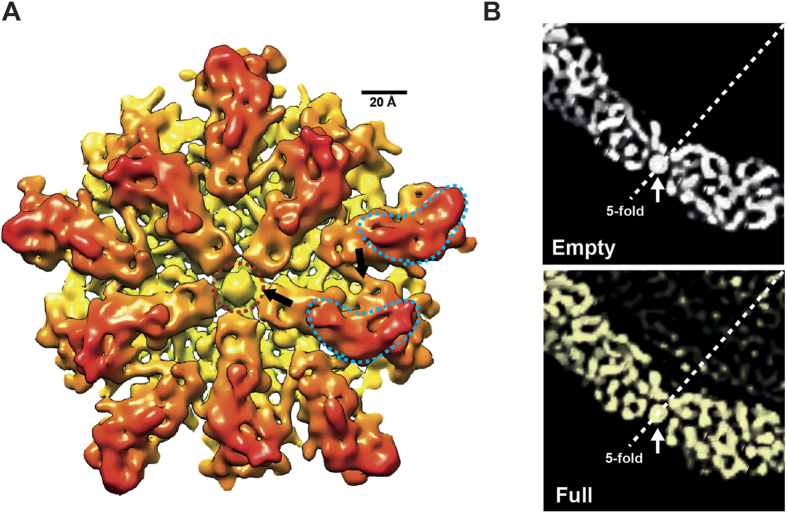
Close-up surface views of the OmRV capsid. (**A**) Close-up view around the 5-fold axis in the empty particle. Arrows indicate regions of interaction between subunits or between a subunit and the obstructed pore. The boomerang-shaped raised ridge domains of subunits and the obstructed pore are outlined with light blue and red dotted lines, respectively. (**B**) Cross-sections of the empty (white) and the full (yellow) particles at the 5-fold axis. White arrows indicate the obstructing plug at the 5-fold axes. The full and empty structures were rendered at an isodensity contour level of 2.1 *σ* and 2.6 *σ*, respectively.

The OmRV particles mainly consist of the 100 kDa major CP proteins that correspond to the C-terminal side of the ORF1 polyprotein (see Supplementary Fig. S2). Ten subunits can be identified around the 5-fold axis and form a plateau area (r = 206 Å in Fig. 3) with a raised ridge (the boomerang-shaped density at r = 215 Å). This raised ridge is also seen in the close-up view of the 5-fold vertex in Fig. 4A. The 20–25 Å pores at the 5-fold axes are obstructed by an ~18 Å density blob in both the full and empty particles (Fig. 4B). The pores are recessed and can be observed in the radial sections of Fig. 3 at 179 Å, 188 Å, and 197 Å (red circles in Fig. 3). Between the plateaus there is a dodecahedron-shaped network of canyons (black areas at r = 206 Å in Fig. 3). Rod-like structures were observed in the radial sections that presumably correspond to main chain and secondary structural elements of the major CP (Fig. 3, especially at r = 188 Å). At this resolution there were only minor differences between the capsids of the full and empty particles, which may be artefacts due to the slight resolution difference in the models.

### Capsid geometry and segmentation

**Figure 5:**
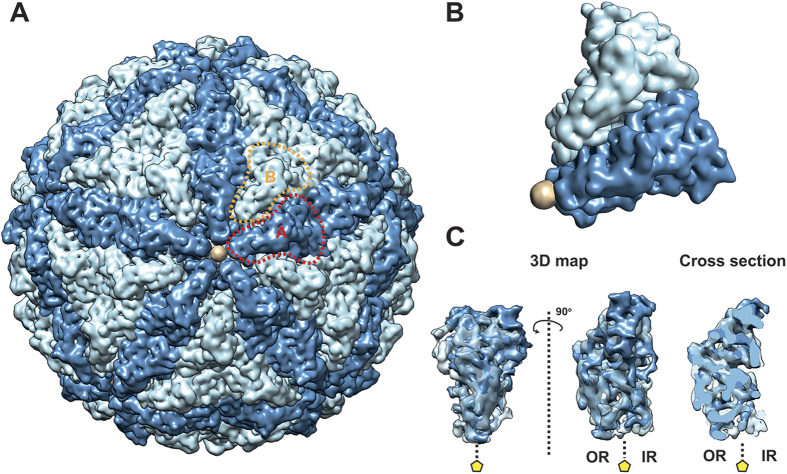
T = 1 capsid geometry of the OmRV particle. (**A**) A and B subunits are coloured dark and light blue, respectively. The obstructing plug is coloured beige. One A-subunit and one B-subunit are outlined by red or orange dotted lines, respectively. (**B**) The asymmetric unit of the OmRV capsid with the pore-blocking density at the 5-fold axis. (**C**) Superimposition of A- and B-subunits in the asymmetric unit of the capsid. The entire 3D maps viewed from the outside of the capsid in the same view as in B (left), a 90-degree rotated view (centre) and a middle cross-section (right) of the monomers are shown. A yellow pentagon indicates a 5-fold axis. OR and IR represent outer and inner regions of the capsids, respectively. The figure was generated using Segger and Chimera. The 3D map of the empty particle was rendered at an isodensity contour level of 2.6 *σ* and segmented.

The icosahedral capsid has a rough but nearly spherical surface with 12 plateau areas composed of 10 subunits around the 5-fold axes (Fig. 5A). The major CPs make up a T = 1 icosahedral shell with 120 subunits (60 dimers) in which the A- and B-subunits form an asymmetric homodimer. The A and B subunits occupy different positions around the 5-fold axis (Fig. 5A,B). The elongated A-subunits are positioned around the 5-fold axis with the B-subunits slightly further away. The tip of the A-subunit is in contact with the plug in the obstructed pore at the 5-fold axis. The overall T = 1 geometry of the OmRV capsid resembles that of IMNV and other totiviruses^12^,^29^,^30^,^31^,^32^. Since the resolution of the cryo-EM structure was not sufficient for automatic segmentation into A and B subunits^30^,^40^, the segmentation was conducted manually as previously described^30^,^31^ (see also *Material and Methods*). The two subunit structures were then aligned and superimposed upon one another (Fig. 5C). The results show, in line with expectations, that the A and B subunits have very similar structures at the resolutions achieved here.

### Secondary structure prediction

**Figure 6:**
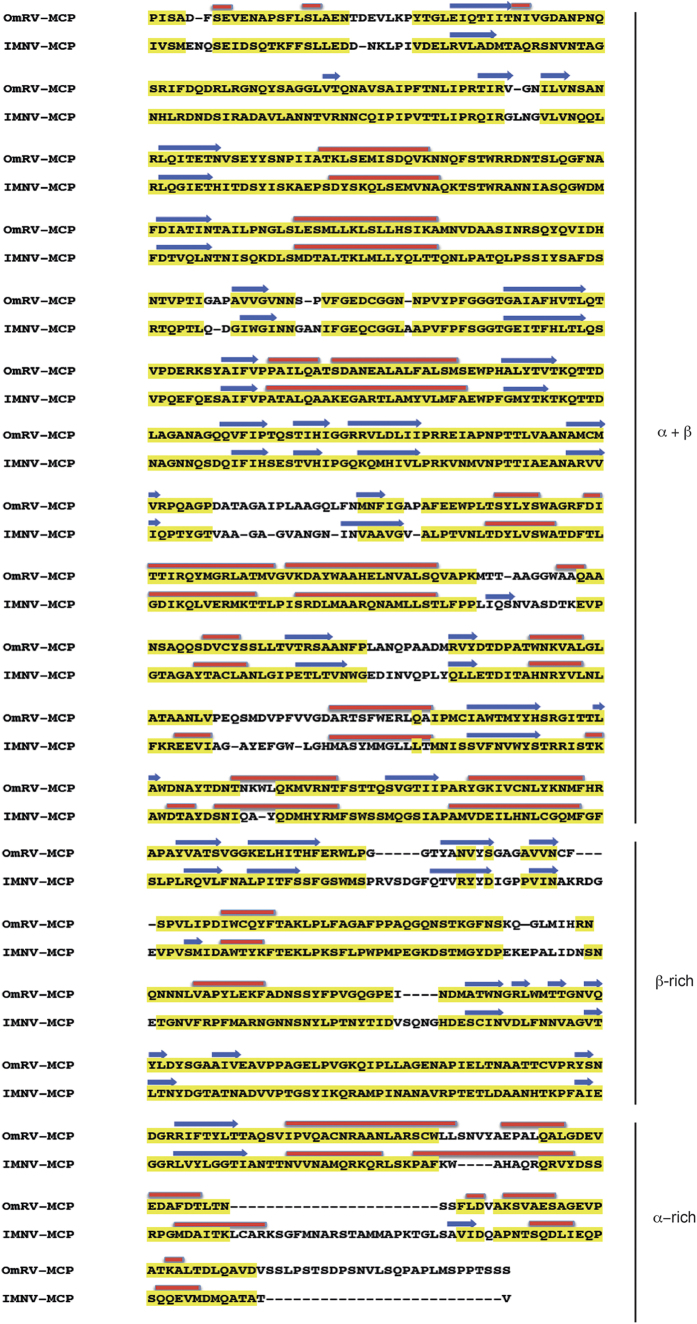
Sequence-based secondary structure prediction. Sequence-predicted secondary structural elements in the aligned amino acid sequences of the major CPs of OmRV and IMNV. Yellow regions were highly conserved sequences as evaluated by T-coffee^58^. The α-helices and β-sheets predicted using PSIPRED^59^ are shown as red rods and blue arrows, respectively.

The sequence-based prediction of secondary structure elements produced identical sequential ordering of the secondary structural elements in the coat proteins of OmRV and IMNV (Fig. 6). This structural similarity was confirmed using a secondary structure-based alignment method (PROMALS3D, see Supplementary Fig. S4). Despite some differences in element order between the different structures, sequence analysis supports the structural division of OmRV into an N-terminal α + β domain with 9–10 major α-helices and several β-sheets, and a C-terminal β-rich domain with 1–2 α-helices and an abundance of β-sheets. The major CPs of OmRV and IMNV are about 200 aa longer than Gag of ScV-L-A. The approximately 200 aa C-terminal extension was predicted to be an α-helix rich domain (α-rich) (Fig. 6). The resolution of the cryo-EM structures does not allow us to assign predicted α-helices accurately in the electron density maps, however, the maps show dominant rod-like structures at positions where helices were predicted by the structure-based helix predictor.

### The capsid structures of totiviruses and totivirus-like viruses

**Figure 7:**
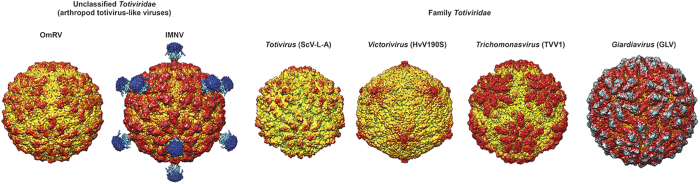
Structural comparison of totivirus, OmRV, and other totivirus-related viruses. Electron density volume map of *Omono River virus* (OmRV, 8.3 Å resolution), *infectious mionecrosis virus* (IMNV, 8.0 Å resolution), *Saccharomyces cerevisiae virus L-A* (ScV-L-A, 8.5 Å resolution), *Heminthosporium victoria virus 190S* (HvV190S, EMDB Accession code: 5726, 7.5 Å resolution), *Trichomonas vaginalis virus 1* (TVV1, EMDB Accession code: 2184, 6.7 Å resolution), and *Giardia lamblia* virus (GLV, EMDB Accession code: 5948, 6.0 Å resolution). The 8.5 Å electron density volume map of ScV-L-A was generated from the crystallographic capsid model (PDB ID: 1M1C) using Chimera^60^. The surface of the structures was coloured according to the distance from the centre of the virion (green < 131 Å, yellow < 193 Å, red < 220 Å, light blue < 250 Å, blue < 280 Å).

The OmRV capsid has a maximum diameter of approximately 44 nm and a T = 1 icosahedral symmetry (Figs 2 and 5A). Figure 7 shows a comparison of all the so far known *Totiviridae* structures. OmRV has a more spherical exterior profile than ScV-L-A. HvV190S and TVV1 and ScV-L-A and HvV190S have smaller capsids while TVV1 has a slightly larger capsid than OmRV. The overall structure of TVV1 resembles that of OmRV with five-fold plateaus and canyons. GLV also has a rather similar spherical capsid but is larger than OmRV (Fig. 7). IMNV shows the closest shape and size to OmRV, but IMNV has large fibre protrusions along the five-fold axes (Fig. 7). To confirm the absence of IMNV-like fibres in the infectious dsRNA-full particles, we collected contrast-enhanced images of OmRV particles using Zernike phase contrast cryo-EM (ZPC-cryoEM) (see Supplementary Fig. S5). The images gave no hint of fibre complexes on the surface of the full or empty particles. The results by Zernike phase contrast cryo-EM agree with conventional defocusing contrast images, and this strongly suggests that OmRV particles lack the IMNV-like fibre complexes.

### The extra raised ridge domain of metazoan totivirus-like viruses

The T = 1 capsid geometry comprises 60 dimers, and is almost universally observed for all dsRNA viruses^10^,^41^. Interestingly, the α + β and β-rich domains of the major CP appear to be conserved among all totiviruses and totivirus-like viruses, albeit with some variation in the sequential and structural order of the secondary structure elements (Fig. 6).

**Figure 8:**
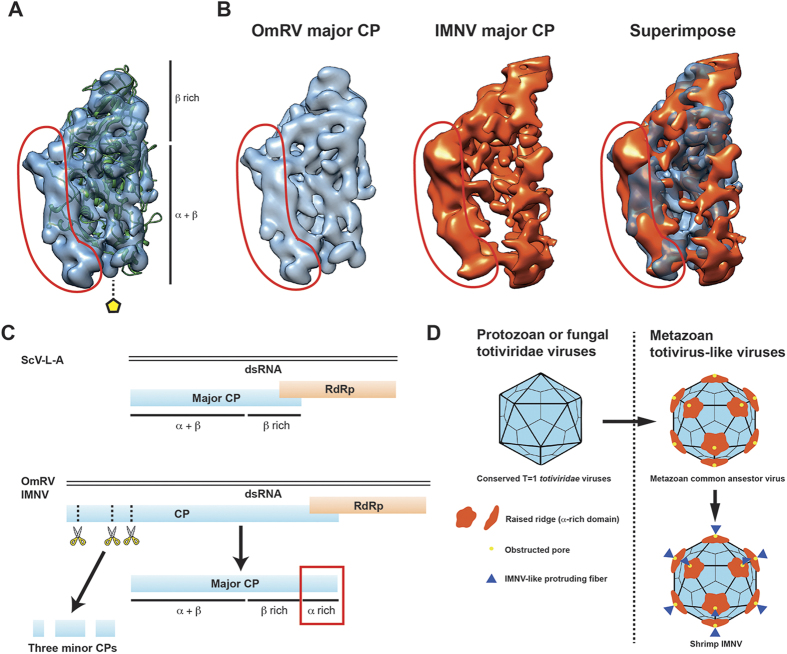
Structural motifs unique to metazoan totvirus-like viruses. (**A**) Superimposition of ScV-L-A capsid protein (green cartoon) on the segmented density of the OmRV major CP (blue surface). The ScV-L-A capsid forms an α + β and a β-rich domain. (**B**) Superimposition of the segmented major CP of OmRV on that of IMNV, shown in light blue and orange, respectively. (**A,B**) The circled red area indicates an extra raised ridge domain of the OmRV major CP that is not present in the ScV-L-A capsid protein. (**C**) Genetic arrangement, expression and post-processing of fungal ScV-L-A and metazoan OmRV and IMNV proteins. The ScV-L-A dsRNA genome encodes a major CP and an RdRp. The major CP adopts a conformation that comprises an α + β and a β-rich domain. The OmRV and the IMNV genomes encode a CP and an RdRp. The CP is post-processed by endogenous cell proteases that divide the CP into three minor CPs and one major CP. The structures of the major CPs of OmRV and IMNV are predicted to comprise an α + β, a β-rich and a C-terminal α-rich domain. (**D**) Graphical model of the acquisition of structural elements in the T = 1 capsid during the evolutionary adaptation of *totiviridae* and totivirus-like viruses to metazoan hosts. All *totiviridae* and totivirus-like viruses adopt conserved T = 1 capsids. Metazoan totivirus-like viruses have an extra raised ridge (α-rich domain) and an obstructed pore in their capsids. Only IMNV has protruding fibres at the 5-fold axes. The α-rich domain and the obstructed pore seem to have been acquired earlier during *totiviridae* evolution, are more deeply conserved in metazoan totivirus-like viruses, and might be fundamental for their survival in metazoan hosts. The IMNV may have acquired extra fibres because of host-specific requirements rather than as a general requirement for metazoan totivirus-like viruses.

The OmRV capsid structure contains a boomerang-shaped raised ridge domain (Figs 3 and 4A). The crystal structure of ScV-L-A Gag was fitted into the segmented cryo-EM structure of OmRV, which shows that the OmRV CP has an extra lobe located on the virion’s exterior surface (Fig. 8A). This domain corresponds to the boomerang-shaped raised ridge. The major CP of OmRV was also superimposed on that of IMNV, and the overall structures of these two CPs matched well with each other (Fig. 8B). Both have a prominent additional external raised ridge (Figs 3 and 8A,B). Based on sequence analyses, including secondary structural prediction (Fig. 6), this extra structure can be attributed to the C-terminal α-rich region of the OmRV CP that follows the conserved α + β and β-rich regions (Fig. 8C). The C-terminal α-rich region was also predicted in the closely related arthropod totivirus-like viruses (DTV, AsTV, and IMNV) (see Supplementary Fig. S4). The intracellularly transmitted TVV1 has a capsid with a corrugated exterior surface and ridges that form plateaus at the 5-fold axes resembling the OmRV and IMNV particles (Fig. 7), but sequence analysis indicated the absence of an α-rich region in the TVV1 CP similar to ScV-L-A Gag (see Supplementary Fig. S4). The CP of TVV1 was also fitted to the crystal structure of ScV-L-A Gag and did not show any extra domain (see Supplementary Fig. S4). Hence, both the sequence and the structure of the TVV1 CP implied that the superficial resemblance of the TVV1 particle to OmRV and IMNV is not due to a homologous extra domain. In intracellular viruses like the TVV1, these ridges have been proposed to be involved in controlling the subcellular localization of particles during division or mating^31^. It has also been observed that dsRNA viruses with multi-layered particles, such as reoviruses and cystoviruses, have an inner capsid with a smooth exterior surface where the conserved T = 1 capsid is completely surrounded by an outer capsid^11^,^42^. This inner capsid is not believed to be important for extracellular transmission.

## Discussion

Metazoan dsRNA viruses have acquired external capsid features during their adaptation to animal hosts, such as the arch-like protruding domain structures in *Picobirnaviridae* or the additional outer capsid in *Reoviridae* viruses^22^. In a similar way, OmRV and IMNV are proposed to have acquired the raised ridge during evolution to gain extracellular transmission capability. The conserved domain is likely important for cell entry in arthropod hosts, perhaps for cell membrane penetration, whereas the additional fibre protrusions of IMNV may be necessary for host-attachment in a manner specific to shrimp and its habitat. The canyon structure created in between the plateaus may harbour a receptor binding site^43^. Such a canyon was first observed in human rhino virus^44^ and the observation led to the proposal of the canyon hypothesis^43^ to explain a mechanism for hiding the host-cell attachment site.

Double-stranded RNA is a strong inducer of the innate immune response of cells and it is believed that the T = 1 capsid of dsRNA viruses evolved as a means for the virus to sequester the genome from the host cell’s nuclease and interferon systems. Part of this strategy involves incorporating RdRp(s) within capsids for replication and synthesis of single-stranded transcripts that function as ribosome templates. In totiviruses, the RdRp is expressed as a RdRp-fused major CP (CP-RdRp) that replaces one or two of the major CPs^45^. The molar ratio of CP-RdRp to major CP was 1:20–35 estimated based on the relative strength of the bands in the SDS-PAGE gel, which indicates that OmRV packages multiple (approximately 3–6) CP-RdRps per virion. Consistent with the RdRp-packaging strategy of totiviruses, the structures of totivirus capsids have 10–20 Å pores, which can function as channels for the extrusion of transcripts into the cytosol of the host cell and for import of nucleotides in the virion^12^,^29^,^31^,^33^. OmRV has a 20–25 Å pore at the five-fold axis that is blocked by an unresolved structural blob (Figs 3 and 4A,B). A similar pore is observed in the IMNV capsid, where this pore is obstructed by the base of the extended fibre complex^32^. The 7611 bp dsRNA of OmRV is densely packed, with an estimated density of 560 mg/mL. This density is higher than that has been observed for any other totivirus (284–473 mg/mL)^12^,^29^,^32^, or dsRNA reovirus (430 mg/mL)^46^. The obstructing plug may be important for stable packing of large amounts of dsRNA. The strategy for RNA synthesis within the capsid is likely to be conserved amongst all *totiviridae* and totivirus-like viruses, and a mechanism that regulates the opening and closing of the pore could provide greater spatial and temporal control.

Minor CPs have been reported in all arthropod-borne totivirus-like viruses. Two 2A-like cleavage sites have been predicted from the amino acid sequences of OmRV^28^, which would fragment the N-terminal part of the ORF1 polypeptide upstream of the CP into three pieces. IMNV has two 2A-like motifs upstream from the major CP, and DTV and AsTV each have one^28^,^47^. MS/MS peptide analysis confirmed the predicted cleavage pattern of OmRV ORF1. N-terminal cleavage products were present in the purified viral fractions and appeared as minor protein bands in SDS-PAGE analyses (see Supplementary Fig. S2). The profiles in the sucrose gradient indicated that the amount of minor CPs correlated strongly with the amount of major CP but less strongly with the amount of dsRNA (Fig. 1C). This result suggests that the minor CPs interact with, or are included in, both dsRNA-full and empty particles. Both IMNV and OmRV have a similarly obstructed pore at the 5-fold axes (Fig. 4B)^32^. Since the obstructed pore is unique to arthropod-borne totivirus-like viruses, one of the minor CPs may compose the obstructing plug. The plug may very well be composed of one of the minor proteins. Minor CP1 was estimated to be present in 12–17 copies per virion, which could correspond to the twelve vertices. Minor CP2 was twice as prevalent and therefore a dimer may form the plug, although this is less likely due to the limited extent of the 18 Å blob. Similarly, the 70 kDa RdRp also seems too large to be attributed to the plug-forming density. There are mushroom-shaped cavities at the 5-fold axes of the HvV190S capsid, which seem to be covered by a protruding cap^30^. The HvV190S capsid proteins are also thought to be post-processed and may produce a small cleavage peptide similar to the arthropod-borne totivirus-like viruses^30^.

In IMNV, the minor proteins are expected to make up the protrusions at the icosahedral 5-fold axes^32^. In contrast, the OmRV particles clearly lack large protrusions, despite the presence of minor CPs in the purified fraction. The fact that almost all full particles of OmRV were infectious to mosquito cells strongly suggests that OmRV does not utilize IMNV-like fibre protrusions for infection. This implies that OmRV either has a different mechanism to enter mosquito cells that is independent of the protrusion structures seen in IMNV^32^ or the plug structure of OmRV has a similar function in the infection chain as the fibre protrusions of IMNV. Both the shrimp and mosquito are arthropods, but their living environments are quite different and it is therefore not surprising that the minor CPs of OmRV and IMNV could have evolved differently to adapt to their hosts. The predicted minor CPs of OmRV and IMNV have different lengths (IMNV: 196 aa, 284 aa, 327 aa; OmRV: 95 aa, 412 aa, 281 aa) and do not exhibit sequence homology. GLV is more closely related to OmRV and IMNV than the other members of the *totiviridae* family, and some of the GLV strains seem to have an extracellular phase^48^. GLV also lacks IMNV-like fibres (although it expresses a small 32-aa peptide) and it was concluded that cell entry by GLV could be mediated by another mechanism^29^. The dsRNA *Baboon reovirus* lacks adhesion fibres at 5-fold vertices, unlike the closely-related *Mammalian orthoreovirus* that exemplifies how regulated gain and loss of minor fibres can be a viable strategy utilized by dsRNA viruses to deal with immune responses, tropism, virulence, transmission, and host range^49^. We suggest that the gain and loss of the minor fibres are also common in *totivirdae* and totivirus-like viruses.

Questions remain about where the OmRV minor proteins are located in the virion and what their functions are. None of them are predicted to be highly hydrophobic nor have an N-terminal glycine suitable for myristoylation, which several other extracellularly transmitting dsRNA viruses have acquired for cell fusion capability^50^,^51^. Another post-transcriptional modification would be required if one of the minor CPs does in fact function as a cell fusion peptide. One of the OmRV minor CPs has a putative dsRNA-binding motif (dsRBM), and similar sequences have been predicted in the other three arthropod totivirus-like viruses^28^. In addition, RaPBV packages a N-terminal cleavage product of the major CP that is putatively involved in genome packing^22^. Thus, the OmRV minor CPs might be located within the virion rather than on the surface and be involved in genome packaging. The OmRV minor CPs were not visible in the current cryo-EM structure.

A model of capsid evolution in *totiviridae* and totivirus-like viruses is shown in Fig. 8D. It is important for viruses with metazoan hosts to acquire an extracellular phase, which requires host-specific cell-binding and cell penetration capabilities. OmRV and IMNV have capsids with two distinguishable features: an extra raised ridge (the α-rich domain) and an obstructed pore at the 5-fold axes. These features seem to be more ancestral than the IMNV-like protruding fibres. The conserved exterior α-rich domain and the canyons between the 5-fold plateaus are probably involved in cell penetration or cell binding. The IMNV-specific fibre may fulfil host- or environment-specific requirements such as shrimp-specific cell-binding. One of the minor CPs of OmRV may act as the plug in the 5-fold pore.

## Methods

### Cell culture and virus propagation

C6/36 *Aedes albopictus* mosquito cells were provided by the Department of Virology, NEKKEN, Nagasaki University. The cells were cultured at 28 °C in minimum essential medium (MEM) supplemented with 10% foetal bovine serum (FBS), 2% nonessential amino acids (NEAA), 100 units/mL penicillin, and 100 μg/mL streptomycin (Gibco, Pen Strep, 15140-122). The original OmRV sample was provided by the National Institute of Infectious Diseases (NIID, Japan). 250 μL of seed OmRV (isolate OmRV-AK4) was inoculated to 80–100% confluent C6/36 cells in 75 cm^2^ flasks. Cells were incubated at 28 °C for 5–7 days until almost all the cells were detached due to the cytopathic effect (CPE).

### OmRV purification and characterization

The method used to purify high concentrations of OmRV was modified from previously described protocols^28^. Infected culture fluid (ICF, 15 mL × 5 flasks) was collected and centrifuged at 10,000 g, 4 °C for 15 min. The ICF was then concentrated using a centrifugal filtration tube (Sartorious stedium, Vivaspin 20, 10,000 molecular weight cut-off) at 6,000 g to a volume of 4 mL. The sample was taken up in 9 mL of 30% sucrose in TNE buffer (20 mM Tris-HCl, 150 mM NaCl, 1 mM EDTA, pH 7.5) in an ultracentrifugation tube (Beckman Coulter, Ultra-Clear Tubes, 344060) before ultracentrifugation at 28,000 rpm (139,190 g), 4 °C for 3 h (Beckman Coulter, Sw40 Ti). The supernatant was discarded and the pellet resuspended in 1 mL of TNE buffer. Resuspended sample was loaded onto 12 mL of a pre-formed 5–50% sucrose gradient in TNE buffer and ultracentrifuged at 18,000 rpm (57,522 g), 4 °C for 18 h (Beckman Coulter, Sw40 Ti), and then fractionated in layers from the top in l mL or 0.5 mL steps. The protein content in the different fractions was checked by measuring absorbance at 280 nm and by SDS-PAGE separation (Invitrogen, NuPAGE). The viral dsRNA of the fractions were extracted using a QIAamp viral RNA Mini kit (Qiagen) and separated by 1.0% agarose gel electrophoresis. The fractions were also negatively stained by 2.0% uranyl acetate and then imaged by transmission electron microscopy (TEM) with an acceleration voltage of 200 kV (JEM2200FS, JEOL). Equal volumes of virus fractions and Mem-Per reagent (Thermo Scientific, B-PERII Bacterial Extraction Reagent) were incubated for 30 min with orbital rotation at room temperature to dissolve large lipidic structures derived from the cells. The sample was passed through a 0.10 μm membrane filter and ultracentrifuged at 28,000 rpm (139,190 g), 4 °C for 3 h (Beckman Coulter, Sw40 Ti). The supernatant was discarded and the pellet resuspended in 1 mL of 100 mM ammonium acetate. The resuspended samples were dialyzed three times in 1 L of 100 mM ammonium acetate, pH 7.5. The final yield of purified viruses ranged from 0.1–0.3 mg from five 15 mL cultures, as determined by measuring absorbance at 280 nm.

### Peptide identification

The four major SDS-PAGE-separated protein bands from OmRV fraction 12 were analysed by MS/MS for peptide identification. The proteins were reduced, alkylated and in-gel digested with trypsin (Roche Applied Science) overnight as described elsewhere (http://www.scilifelab.se/facilities/bioanalytical-proteomics/). The digested products were extracted by sonication in 5% formic acid and 60% acetonitrile. The peptides were separated by reversed-phase chromatography on a C18-column in a 15 mL 0.1% formic acid acetonitrile gradient (A: 0% and B: 99.9% acetonitrile), electrosprayed on-line to a Q Exactive Plus mass spectrometer (Thermo Finnigan) and sequentially analysed by MS/MS tandem spectrometry. Obtained data were analysed by Proteome Discover 1.4 (Thermo Fisher Scientific) with an in-house FASTA database containing proteins from OmRV, and other possible contaminations such as serum *Bos Taurus, Escherichia coli* and *Homo sapiens* downloaded from UniprotKB. The search criteria for protein identification were set to at least two matching peptides at a 95% confidence level per protein. Proteins were identified, and scores calculated, by SEQUEST^52^.

### Infectivity assay

The concentration of the purified OmRV fractions was adjusted to 0.28 mg/mL and then diluted 1000 times in TNE buffer. 100 μL of the OmRV fractions were serially diluted and inoculated into 80–100% confluent C6/36 cells in 12-well plates. The collected samples were titrated using plaque-forming assays as described previously^28^. Briefly, 100 μL each of the serially diluted ICFs were inoculated into 12 well plates and incubated for 1 h at 28 °C. After removing and washing the ICFs, 1 mL of 1.0% agarose-containing medium was overlaid on the infected C6/36 cells. Three days post-inoculation, 1.0% agarose supplemented with 0.85% NaCl and 0.013% neutral red was loaded on the plate. The plaques were counted the following day.

### Nanoparticle tracking analysis (NTA)

An NTA system illuminates particles using laser light and a microscope camera records a movie of the diffracted light. Particle sizes can be estimated based on diffusion rates between consecutive frames of the movie for sphere-like objects assuming Brownian motion. Particles in the range of 30 to 1000 nm and concentrations in the range of 10^7^ to 10^9^ particles/mL can be tracked. This method has been used for counting the number of virus particles in solution^53^. The purified fraction 22–26 (0.28 mg/mL) was diluted 1 in 10 in TNE buffer to ensure a sufficient volume of sample for NTA measurements. The size distribution was measured by a nanoparticle tracking system (Nanosight, LM14) with a 405 nm laser. 300 μL of the sample was applied and tracked for 20 seconds, and was measured three times. Analysis was performed using version 3.0 of the NTA software (Nanosight). The detection limit of NTA is 10^7^ particles/mL. The 10 times diluted sample resulted in too few tracked particles, so the number of particles in the original sample was expected to be less than 10^8^ particles/mL (10^7^ particles/mL × 10 times dilution) owing to the detection limit.

### Cryo-EM, single particle reconstruction, and data analysis

Purified and further concentrated OmRV (1.43 mg/mL) was embedded in vitreous ice and examined at liquid nitrogen temperature with a cryo-EM (JEM2200FS; JEOL) operated at an accelerating voltage of 200 kV and at a nominal magnification of × 50,000. An omega-type energy filter was used to obtain a zero loss electron beam (20 eV slit width). Images were recorded on a 4k × 4k charge-coupled device (CCD; TVIPS) with an electron dose of ∼20 e^−^/Å^2^ with applied underfocus values of approximately 1–3 μm and at pixel size of 1.88 Å/pixel. EMAN2 was used for selecting individual viral particles^54^. Empty and dsRNA-full particles were boxed separately. Contrast transfer function (CTF) and amplitude corrections were automatically performed on boxed particles by e2ctf.py (EMAN2) and then manually verified by adjusting defocus and B-factor values. Several random icosahedral initial models were calculated by 15 class-averaged OmRV particle images, each calculated using 300 raw particle images. A total of 12,207 empty and 6,224 full particles were aligned and classified to reconstruct the two 3D structures using EMAN2 and RELION^54^,^55^. One calculated random model was selected and used for reference-based iterative refinement using icosahedral symmetry by EMAN2 to obtain an initial 3D reconstruction. After 10 iterative steps of 3D refinement, the first refined model was computed from empty OmRV particles and used for 3D refinement using RELION. In RELION, CTF and amplitude corrections of images were automatically performed by ctffind3^56^. Good particles were extracted from good 2D class-averaged images and used for maximum likelihood-based 3D reconstruction using RELION^55^. The final resolution of the empty OmRV model was estimated to be 8.3 Å (FSC cutoff 0.143). A full particle was reconstructed from a particle set of dsRNA-full particles by using the final empty OmRV structure as an initial model using RELION. Segmentation analyses of OmRV and IMNV capsid subunits were conducted manually with the assumption that the two chemically identical capsid protein subunit types should roughly share the same shape. The two subunits were separately extracted from the overall structure by masking away all density clearly belonging to neighbouring subunits. The structures of the two subunits were then superimposed and the final subunit structure was determined by using the identity between the two subunits using Chimera as previously described^30^,^31^. The 3D volumes and cross sections of OmRV, IMNV and other *totiviridae* viruses were visualized by Chimera and Segger software. The 3D maps were rendered at an isodensity contour level of 2.6 *σ* and 2.1 *σ* (equivalent to numerical map values of 0.0900 and 0.0938) in empty and full particles. in Chimera. The radial sections were generated by bsoft (bradsec)^57^.

### Sequence alignment and secondary structure prediction

The cleavage sites of the OmRV and IMNV CPs were predicted in previous articles^28^,^47^; MS/MS identification of the OmRV major CP in this study agreed with those predictions (see Supplementary Fig. S2). The predicted amino acid sequences of the major CPs were aligned by the T-coffee multiple sequence alignment server^58^. Additionally, the sequences were applied to the PSIPRED protein sequence analysis workbench for predicting secondary structures^59^.

## Additional Information

**Accession codes:** The 3D density maps of the reconstructed dsRNA-full and empty OmRV have been deposited in the Electron Microscopy Data Bank (EMDB) at the European Bioinformatics Institute with accession codes EMD-3350 (empty) and EMD-3351 (full), respectively.

**How to cite this article**: Okamoto, K. *et al*. The infectious particle of insect-borne totivirus-like Omono River virus has raised ridges and lacks fibre complexes. *Sci. Rep.* **6**, 33170; doi: 10.1038/srep33170 (2016).

## Supplementary Material

Supplementary Information
