# Supplementary material for: The infectious particle of insect-borne totivirus-like Omono River virus has raised ridges and lacks fibre complexes
Source: Sci Rep. 2016 Sep 12;6:33170. doi: 10.1038/srep33170 (PMC5018817; doi:10.1038/srep33170)
Supplement: Supplementary Information [file srep33170-s1.pdf]

The infectious particle of insect-borne totivirus-like Omono River virus has raised ridges and lacks fibre complexes

*Kenta Okamoto[1], Naoyuki Miyazaki[2], Daniel S. D. Larsson[1], Daisuke Kobayashi[3], Martin Svenda[1], Kerstin Mühlig[1], Filipe R.N.C. Maia[1], Laura H. Gunn[1], Haruhiko Isawa[3], Mutsuo Kobayashi[3], Kyoko Sawabe[3], *Kazuyoshi Murata[2], and Janos Hajdu[1]

1. The Laboratory of Molecular Biophysics, Department of Cell and Molecular biology, Uppsala University, Sweden
2. National Institute for Physiological Sciences (NIPS), Japan
3. Department of Medical Entomology, National Institute of Infectious Diseases (NIID), Japan

*Corresponding author1: Kenta Okamoto e-mail: kenta.okamoto@icm.uu.se

*Corresponding author2: Kazuyoshi Murata e-mail: kazum@nips.ac.jp

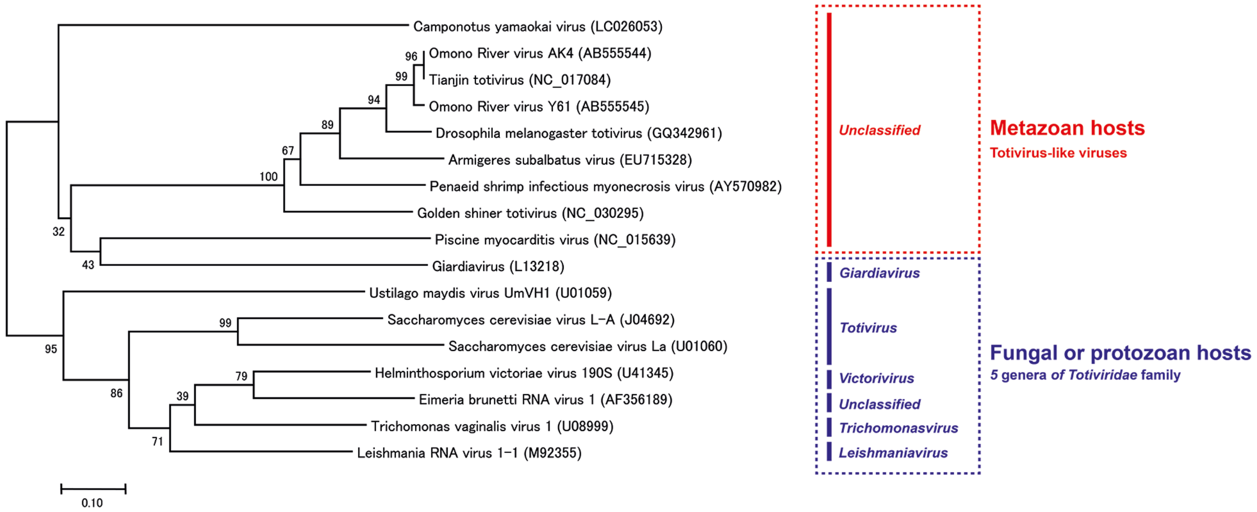
Camponotus yamaokai virus (LC026053)
Omono River virus AK4 (AB555544)
Tianjin totivirus (NC_017084)
Omono River virus Y61 (AB555545)
Drosophila melanogaster totivirus (GQ342961)
Armigeres subalbatus virus (EU715328)
Penaeid shrimp infectious myonecrosis virus (AY570982)
Golden shiner totivirus (NC_030295)
Piscine myocarditis virus (NC_015639)
Giardiavirus (L13218)
Ustilago maydis virus UmVH1 (U01059)
Saccharomyces cerevisiae virus L-A (J04692)
Saccharomyces cerevisiae virus La (U01060)
Helminthosporium victoriae virus 190S (U41345)
Eimeria brunetti RNA virus 1 (AF356189)
Trichomonas vaginalis virus 1 (U08999)
Leishmania RNA virus 1-1 (M92355)
96
99
94
89
67
100
32
43
99
95
86
79
39
71
0.10
Unclassified
Metazoan hosts
Totivirus-like viruses
Giardiavirus
Totivirus
Victorivirus
Unclassified
Trichomonasvirus
Leishmaniavirus
Fungal or protozoan hosts
5 genera of Totiviridae family

Fig. S1 Phylogenetic relationships among five genera of *totiviridae* viruses and unclassified *totiviridae* viruses inferred from RdRp amino acid sequences. The GenBank or the RefSeq accession numbers are shown in the figure. The unclassified *totiviridae* viruses above the Giardia virus are called totivirus-like viruses including OmRV, IMNV, PMCV, AsMV, and DTV and are infectious to metazoan hosts such as mosquitoes, shrimps, and fish. The five Totivirus genera and the unclassified viruses below Giardia virus are infectious to fungal or protozoan hosts except plant Ustilago maydis virus (UmVH1). The sequences were aligned using ClustalW[1]. The phylogenetic tree was built using a neighbour-joining algorithm in ClustalW.

A

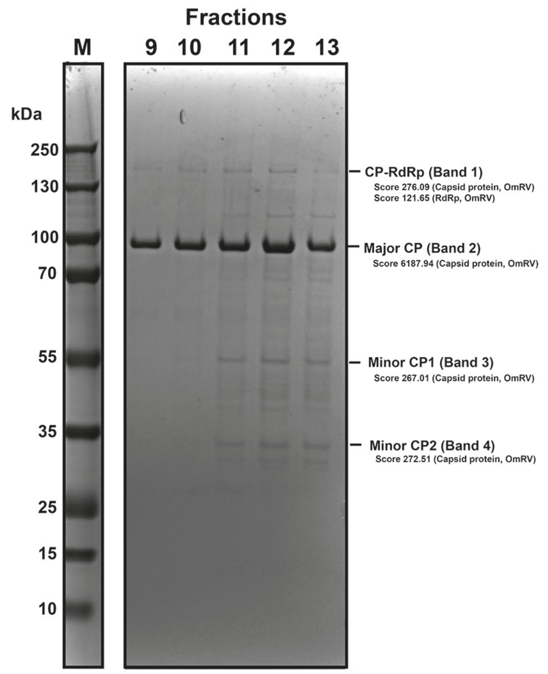

B

N-term
MPGYSANYREGPVIVPSGDQVVTSPTSHGRSKGVSSELLTVGGQTYKAIASNEVV
KSGQAKGEQKFFDENAKIVLLGTFPFKKDLTKEGVEPNPGPVVSDPYANTIYGPL
PTHKEAAINLGEQPSAPVKQRPKRHAVVDRVYKTLTLKNGILVTKVVIAGVATYI
VYKVGSEVWSWYAAITKPLLGVQTAVKEEYHTLQHKYDYQWYDYFCPMCLLSKYI
ANSGVNGTDAEHHNHVMHALNGNIDCDSSVFGNNFNITTSPQTLTMSGPLAPGKY
QTTLTVQALIGGTGVVVGTVTFAGKTVAYQVFDDSFASFDLGTVTVSASTAPSVI
WTGSTGATLTMAVNIICKPITPTSVAISGQPIWTTPYAPAQAVMTVPAVAKALKN
TQRAADLSTRNKTRHGSNNAISVLNQLATQIDVEDQRLPIYKIEGEREGTHEPSF
SCQLTFRGMTKSAFGKRTKHEAKTAAASLILEELTRKSNSTWPADAKLTLSGWVR
DLTEEGIEPNPGPIANEDGAECWDPEELKMLQAMMAREQQKVLPKMIDDPKLVLD
LDEMQQLAPQESCNKTVSVDEYQEHVRHMSNYSGAVGEAFAVVRWYLLNETTNRF
APLVVMDDSMDEPIATPVDVSPAKRPSGVSRRSDKEEEKPKKPPKKEKIVPAPQP
PKTQEQLDEERKFAMQRIADRIKSDPIKLLTWIKRPTSTKFKREVLSLAFDIPHI
NSKPSLDQYETIVYCYFNDIPDDELTIMLLQYDRKWTSLSTDCKLSILSACNVRA
SLEAAKAHNKLMHAYNGNPISADFSEVENAPSFLSLAENTDEVLKPYTGLEIQTI
ITNIVGDANPNQSRIFDQDRLRGNQYSAGGLVTQNAVSAIPFTNLIPRTIRVGNI
LVNSANRLQITETNVSEYYSNPIIATKLSEMISDQVKNNQFSTWRRDNTSLQGFN
AFDIATINTAILPNGLSLESMLLKLSLLHSIKAMNVDAASINRSQYQVIDHNTVP
TIGAPAVVGVNNSPVFGEDCGGNNPVYPFGGGTGAIAFHVTLQTVPDERKSYAIF
VPPAILQATSDANEALALFALSMSEWPHALYTVTKQTTDLAGANAGQQVFIPTQS
TIHIGGRRVLDLIIPRREIAPNPTTLVAANAMCMVRPQAGPDATAGAIPLAAGQL
FNMNFIGAPAFEEWPLTSYLYSWAGRFDITTIRQYMGRLATMVGVKDAYWAAHEL
NVALSQVAPKMTTAAGGWAAQAANSAQQSDVCYSSLLTVTRSAANFPLANQPAAD
MRVYDTDPATWNKVALGLATAANLVPEQSMDVPFVVGDARTSFWERLQAIPMCIA
WTMYYHSRGITTLAWDNAYTDNTNKWLQKMVRNTFSTTQSVGTIIPARYGKIVCN
LYKNMFHRAPAYVATSVGGKELHITHFERWLPGGTYANVYSGAGAVVNCFSPVLI
PDIWCQYFTAKLPLFAGAFPPAQGQNSTKGFNSKQGLMIHRNQNNNLVAPYLEKF
ADNSSYFPVGQGPEINDMATWNGRLWMTTGNVQYLDYSGAAIVEAVPPAGELPVG
KQIPLLAGENAPIELTNAATTCVPRYSNDGRRIFTYLTTAQSVIPVQACNRAANL
ARSCWLLSNVYAEPALQALGDEVEDAFDTLTNSSFLDVAKSVAESAGEVPATKAL
TDLQAVDVSSLPSTSDPSNVLSQPAPLMSPPTSSS C-term

C

ORF1 (CP)

N-term
MPGYSANYREGPVIVPSGDQVVTSPTSHGRSKGVSSELLTVGGQTYKAIASNEVV
KSGQAKGEQKFFDENAKIVLLGTFPFKKDLTKEGVEPNPGPVVSDPYANTIYGPL
PTHKEAAINLGEQPSAPVKQRPKRHAVVDRVYKTLTLKNGILVTKVVIAGVATYI
VYKVGSEVWSWYAAITKPLLGVQTAVKEEYHTLQHKYDYQWYDYFCPMCLLSKYI
ANSGVNGTDAEHHNHVMHALNGNIDCDSSVFGNNFNITTSPQTLTMSGPLAPGKY
QTTLTVQALIGGTGVVVGTVTFAGKTVAYQVFDDSFASFDLGTVTVSASTAPSVI
WTGSTGATLTMAVNIICKPITPTSVAISGQPIWTTPYAPAQAVMTVPAVAKALKN
TQRAADLSTRNKTRHGSNNAISVLNQLATQIDVEDQRLPIYKIEGEREGTHEPSF
SCQLTFRGMTKSAFGKRTKHEAKTAAASLILEELTRKSNSTWPADAKLTLSGWVR
DLTEEGIEPNPGPIANEDGAECWDPEELKMLQAMMAREQQKVLPKMIDDPKLVLD
LDEMQQLAPQESCNKTVSVDEYQEHVRHMSNYSGAVGEAFAVVRWYLLNETTNRF
APLVVMDDSMDEPIATPVDVSPAKRPSGVSRRSDKEEEKPKKPPKKEKIVPAPQP
PKTQEQLDEERKFAMQRIADRIKSDPIKLLTWIKRPTSTKFKREVLSLAFDIPHI
NSKPSLDQYETIVYCYFNDIPDDELTIMLLQYDRKWTSLSTDCKLSILSACNVRA
SLEAAKAHNKLMHAYNGNPISADFSEVENAPSFLSLAENTDEVLKPYTGLEIQTI
ITNIVGDANPNQSRIFDQDRLRGNQYSAGGLVTQNAVSAIPFTNLIPRTIRVGNI
LVNSANRLQITETNVSEYYSNPIIATKLSEMISDQVKNNQFSTWRRDNTSLQGFN
AFDIATINTAILPNGLSLESMLLKLSLLHSIKAMNVDAASINRSQYQVIDHNTVP
TIGAPAVVGVNNSPVFGEDCGGNNPVYPFGGGTGAIAFHVTLQTVPDERKSYAIF
VPPAILQATSDANEALALFALSMSEWPHALYTVTKQTTDLAGANAGQQVFIPTQS
TIHIGGRRVLDLIIPRREIAPNPTTLVAANAMCMVRPQAGPDATAGAIPLAAGQL
FNMNFIGAPAFEEWPLTSYLYSWAGRFDITTIRQYMGRLATMVGVKDAYWAAHEL
NVALSQVAPKMTTAAGGWAAQAANSAQQSDVCYSSLLTVTRSAANFPLANQPAAD
MRVYDTDPATWNKVALGLATAANLVPEQSMDVPFVVGDARTSFWERLQAIPMCIA
WTMYYHSRGITTLAWDNAYTDNTNKWLQKMVRNTFSTTQSVGTIIPARYGKIVCN
LYKNMFHRAPAYVATSVGGKELHITHFERWLPGGTYANVYSGAGAVVNCFSPVLI
PDIWCQYFTAKLPLFAGAFPPAQGQNSTKGFNSKQGLMIHRNQNNNLVAPYLEKF
ADNSSYFPVGQGPEINDMATWNGRLWMTTGNVQYLDYSGAAIVEAVPPAGELPVG
KQIPLLAGENAPIELTNAATTCVPRYSNDGRRIFTYLTTAQSVIPVQACNRAANL
ARSCWLLSNVYAEPALQALGDEVEDAFDTLTNSSFLDVAKSVAESAGEVPATKAL
TDLQAVDVSSLPSTSDPSNVLSQPAPLMSPPTSSS C-term

ORF2 (RdRp)

N-term
MSTPGDILTTDYRVKSDLAVTRLRIKDFIARIDPNIVKFMNQHLCHLDQTILANL
CIWTQLWGLRHLVTLHSLGMLNDIDTYATKMAKVSSFAKRFPFETDNAKQRWCEV
NTLTGYMQNDIGTFDYDKEFESLATGGNEHPEWWKRKFEEKVRDLMTFQEAPEFV
SFETYVKDGYWLTSGSSSVGKVEWSYDGDTGKFKARKNMLVDLYTKEEIYQIAID
WDGELMNRVFIKDELAKRRLAVASNIEAYLNQAYLLYLFGHGFKNYKYITLDEKP
NETHKRNCRLIKLLKEGSYALPFDFKGFDRQPTTDEIKTIIKRVIDLVSPRVPAS
HRRLFNTIAFKNIACYDKNYLYSPLTKRTVKQTGGLPSGIRPTSLIGNLWNMIAT
DIARDVTKDILGQDYIQEIALKGDDTYIVAPNFFVCLVFRYAYQSINAVGENSKF
GIMQNACEFLRTEISSSGVRGWTNRAIPSVTQRKPWNPEPWTENQQVQTIANNIY
LLERRCKQDCTLLHMANKIKWSKMMHQSYLWLHLPKHHGGFGIYEWSGWLPDCKL
PLTQPPVFDVANLNPSSVDLSWYSLSDSEKRAYQKVEFSSKIAANDIPGPAKHVM
RSYIVALRACKPEWSKTLVNFKPIPLISGPTYTNPFWPHPRIKTDTTSNTNGFPQ
LSEFVRQHQIAKRAKIPVPPLRDMIAKHYPSAYKVMNRCELQGWHRTDSINIACG
KIPTEPLKILNPILAAWVQQIVLNAGLLKWSGRKNIANRLYAATTLAVHHIQCEG
GASMYAF C-term

Fig. S2 Identification of OmRV proteins in the purified fractions. A) The four protein bands that were analysed using tandem-MS spectrometry. The proteins were identified and scores calculated by SEQUEST[2]. Each lane number represents a 1 mL fraction from a 14 mL 5-50% sucrose density gradient numbered from top to bottom. B) Detected peptides from the major and minor OmRV proteins. The full amino acid sequence of ORF1 is shown. Peptides only detected in the minor CP1 and minor CP2 bands (Band 3 and Band 4), and not detected in the major CP band (Band 2), are shown in red. Peptides detected in the major CP band (Band 2) are shown in blue. C) Detected peptides of the CP (ORF1) and the RdRp (ORF2) from Band 1. The detected peptides are shown in red.

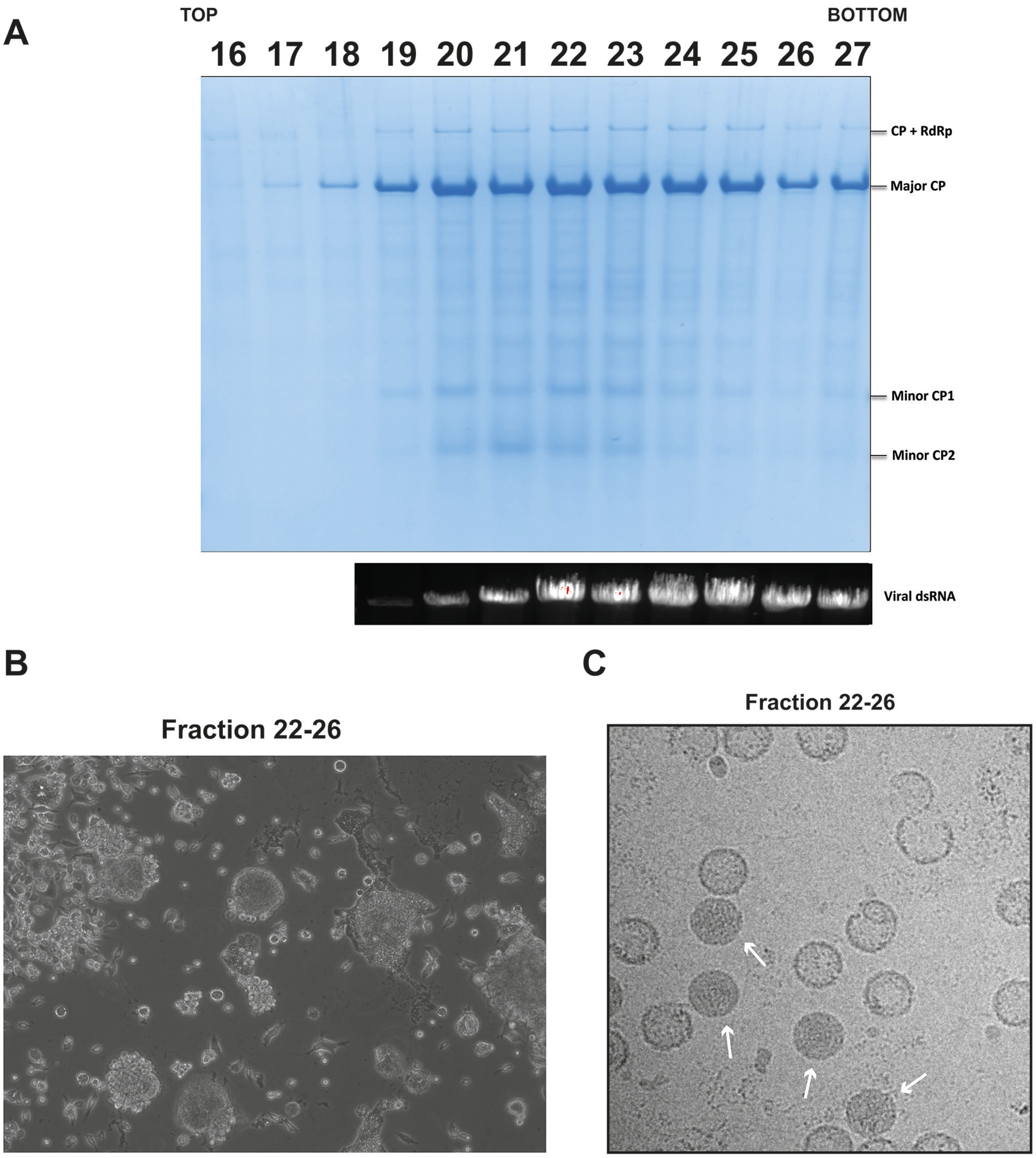
A
TOP
BOTTOM
16 17 18 19 20 21 22 23 24 25 26 27
CP + RdRp
Major CP
Minor CP1
Minor CP2
Viral dsRNA
B
Fraction 22-26
C
Fraction 22-26

Fig. S3 Protein and dsRNA compositions and the infectivity titre in purified OmRV fractions. A) SDS-PAGE and agarose gel electrophoresis of the protein- and dsRNA-containing fractions from the 14 mL 5-50% sucrose density gradient. Each lane number represents a 500 μL fraction numbered from top to bottom. B) Cytopathic effects on C6/36 mosquito cells three days post inoculation of pooled fraction 22-26. The sample was diluted 10^5 times before inoculation. C) Raw cryo-EM images of pooled fraction 22-26 that was used for the plaque assay. The dsRNA-full particles are indicated by arrows.

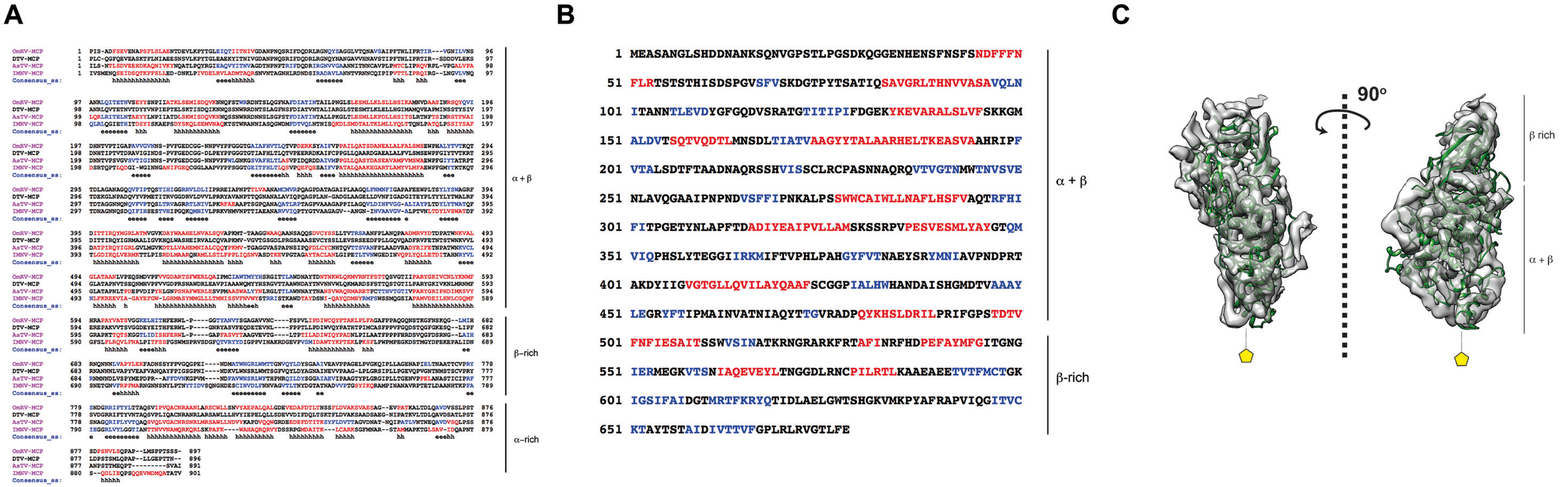
A
α + β
β–rich
α–rich
B
1 MEASANGLSHDDNANKSQNVGPSTLPGSDKQGGENHENSFNSFSNDFFFN
51 FLRTSTSTHISDSPGVSFVSKDGTPYTSATIQSAVGRLTHNVVASAVQLN
101 ITANNTLEVDYGFGQDVSRATGTITIPIFDGEKYKEVARALSLVFSKKGM
151 ALDVTSQTVQDTLMNSDLTIATVAAGYYTALAARHELTKEASVAAHRIPF
201 VTALSDTFTAADNAQRSSHVISSCLRCPASNNAQRQVTVGTNMWTNVSVE
251 NLAVQGAAIPNPNDVSFFIPNKALPSSWWCAIWLLNAFLHSFVAQTRFHI
301 FITPGETYNLAPFTDADIYEAIPVLLAMSKSSRPVPESVESMLYAYGTQM
351 VIQPHSLYTEGGIIRKMIFTVPHLPAHGYFVTNAEYSRYMNIAVPNDPRT
401 AKDYIIGVGTGLLQVILAYQAAFSCGGPIALHWHANDAISHGMDTVAAAY
451 LEGRYFTIPMAINVATNIAQYTTGVRADPQYKHSLDRILPRIFGPSTDTV
501 FNFIESAITSSWVSINATKRNGRARKFRTAFINRFHDPEFAYMFGITGNG
551 IERMEGKVTSNIAQEVEYLTNGGDLRNCPILRTLKAAEAEETVTFMCTGK
601 IGSIFAIDGTMRTFKRYQTIDLAELGWTSHGKVMKPYAFRAPVIQGITVC
651 KTAYTSTAIDIVTTVFGPLRLRVGTLFE
α + β
β-rich
C
90°
β rich
α + β

Fig. S4 PSIPRED-based secondary structure prediction and sequence alignment. A) PROMALS3D sequence alignment of arthropod totivirus-like virus (OmRV, DTV, AsTV, IMNV) major CPs (MCP) using PSIPRED-based secondary structure predication[3]. The red and blue sequences indicate the α-helices and β-sheets predicted by PSIPRED protein sequence analyses. The predicted consensus α-helices (h) and β-sheets (e) are indicated. All arthropod totivirus-like viruses have a conserved C-terminal α-helix-rich domain not found in fungal and protozoan totiviruses. B) PSIPRED-based secondary structure predication of TVV1. The red and blue sequences indicate α-helices and β-sheets, respectively, as predicted by PSIPRED protein sequence analyses. TVV1 does not have the C-terminal α-helix-rich domain found in arthropod totivirus-like viruses. C) Superimposition of ScV-L-A capsid protein (green cartoon) on the segmented density of the TVV1 CP (grey surface). The ScV-L-A capsid forms an α + β and a β-rich domain. The orientation of the TVV1 CP is same as those of the MCPs in Fig. 8A and 8B (right). A 90-degree rotated view (left). A yellow pentagon indicates a 5-fold axis.

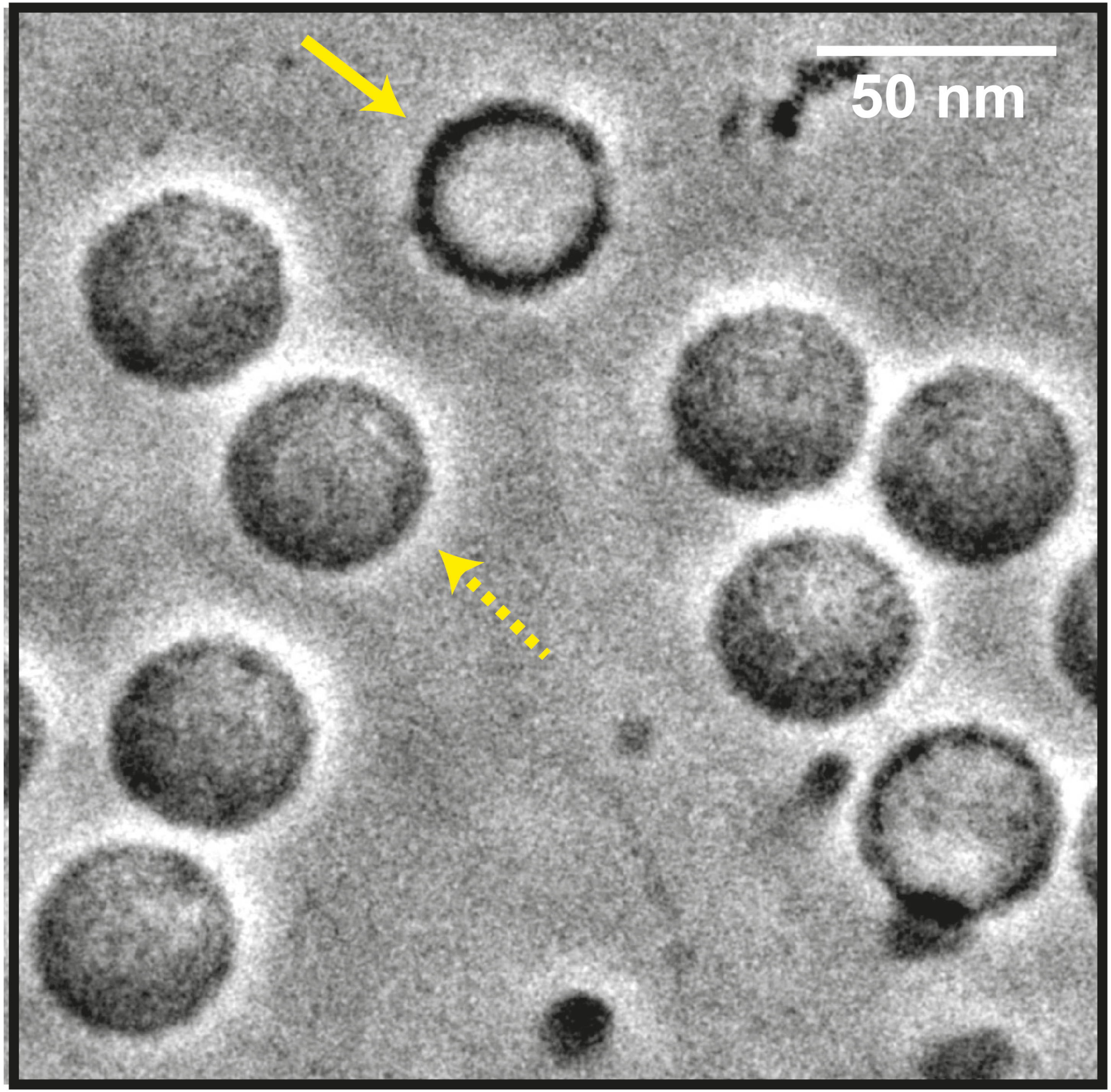
50 nm

Fig. S5 Zernike phase contrast cryo-EM image of ice-embedded OmRV. Apart from being observed in focus using the Zernike phase plate[4], the image was taken under the same conditions as the defocus contrast image (Fig. 2A). Solid and dotted arrows indicate empty and full virus particles, respectively.

References

1 Larkin, M. A. *et al.* Clustal W and Clustal X version 2.0. *Bioinformatics* **23**, 2947-2948 (2007).

2 Eng, J. K., McCormack, A. L. & Yates, J. R. An approach to correlate tandem mass spectral data of peptides with amino acid sequences in a protein database. *Journal of the American Society for Mass Spectrometry* **5**, 976-989 (1994).

3 Pei, J. & Grishin, N. V. PROMALS3D: multiple protein sequence alignment enhanced with evolutionary and three-dimensional structural information. *Methods in molecular biology* **1079**, 263-271 (2014).

4 Murata, K. *et al.* Zernike phase contrast cryo-electron microscopy and tomography for structure determination at nanometer and subnanometer resolutions. *Structure* **18**, 903-912 (2010).
